# Prognostic Value of Mandard and Dworak Tumor Regression Grading in Rectal Cancer: Study of a Single Tertiary Center

**DOI:** 10.1155/2014/310542

**Published:** 2014-03-04

**Authors:** Marisa D. Santos, Cristina Silva, Anabela Rocha, Eduarda Matos, Carlos Nogueira, Carlos Lopes

**Affiliations:** ^1^Department of Surgery, Digestive Surgery Service, Hospital de Santo António, Largo Professor Abel Salazar, 4099-003 Porto, Portugal; ^2^Department of Community Health, Instituto de Ciências Biomédicas Abel Salazar, Rua Jorge Viterbo Ferreira No. 228, 4050-313 Porto, Portugal; ^3^Department of Pathology, Pathological Anatomy Service, Hospital de Santo António, Largo Professor Abel Salazar, 4099-003 Porto, Portugal; ^4^Department of Pathology and Molecular Immunology, Instituto de Ciências Biomédicas Abel Salazar, Rua Jorge Viterbo Ferreira No. 228, 4050-313 Porto, Portugal

## Abstract

*Goal*. To evaluate the prognostic value of Mandard and Dworak grading systems regarding neoadjuvant chemoradiotherapy (CRT) response on rectal cancer. *Materials and Methods*. We queried our center's database for patients with colo rectal cancer with locally advanced rectal cancer (LARC) who received neoadjuvant CRT followed by total mesorectum excision (TME) between 2003 and 2011. After excluding 18 patients from the initial query the remaining 139 were reassessed for disease recurrence and survival; the specimens' slides were reviewed and classified according to two tumor regression grading (TRG) systems: Mandard and Dworak. Based on these TRG scores, two patient groups were created: patients with good response versus patients with bad response (Mandard TRG1+2 versus Mandard TRG3+4+5 and Dworak TRG4+3 versus Dworak TRG2+1+0). Overall survival (OS), disease-free survival (DFS), and disease recurrence were then evaluated. *Results*. Mean age was 64.2 years and median follow up was 56 months. No significant survival difference was found when comparing patients with Dworak TRG 4+3 versus Dworak TRG2+1+0 (*P* = 0.10). Mandard TRG1+2 presented with significantly better OS and DFS than Mandard TRG3+4+5 (OS *P* = 0.013; DFS *P* = 0.007). *Conclusions*. Mandard system provides higher accuracy over Dworak system in predicting rectal cancer prognosis when neoadjuvant CRT is applied for tumor regression.

## 1. Introduction

Improved outcome in the treatment of locally advanced rectal cancer (LARC) is related to the introduction of total mesorectal excision (TME) and neoadjuvant treatment [1–3].

In locally advanced rectal cancer (LARC) the use of neoadjuvant chemoradiotherapy (CRT) reduces locoregional recurrence and can lead to better prognosis depending on the tumor regression grade. Rectal cancer prognosis appears to be related to neoadjuvant CRT response [4–6].

After curative surgery with TME, tumor extension through the rectal wall (pT), spreading to the regional lymph nodes (pN) and the circumferential resection margin (CRM) constitute the main criteria to estimate prognosis in rectal carcinoma patients [7]. In LARC, chemoradiotherapy applied before surgery may change the pathologic stage and CRM of the resected specimen. Several studies have demonstrated that clinical outcome depends not only on the initial stage of the tumor, but also on the CRT-induced tumor response which varies among individual patients [8].

Tumor response to neoadjuvant CRT can induce cytoreduction and downstaging of the lesion and can also cause histological changes which can be assessed by tumor regression systems, which in turn offer another method for evaluating tumor regression.

Tumor regression can range from zero evidence of treatment efficacy to a complete response (ypCR) with no viable tumor cells identified. It is well established that patients with pCR after chemoradiation have better long-term outcomes than those without pCR [5, 9, 10]. Complete response, however, accounts for less than one third of the patients, and the majority of patients present either partial or no response at all. The prognostic value of partial or near complete response is an important topic and research is underway [6, 11].

In order to quantify neoadjuvant CRT response, several grades can be used, being particularly important in situations where the pathological response is not complete [12–18]. There is no consensual regression grading system for pathologists who are presented with resected tumor specimens following neoadjuvant chemoradiotherapy. Most have 3 to 5 levels, allowing group creation according to the responses. This lack of consensus impeaches clinical management and leaves clinicians without a uniform scoring regression system that could guide their decisions.

The value of tumor regression grading systems as an independent prognostic factor for disease-free survival has been demonstrated in several studies [6, 19–21]. The present study aims to evaluate the accuracy of Mandard and Dworak systems in rectal cancer neoadjuvant chemoradiotherapy (CRT) as a prognostic factor, mainly for patients who achieved a near complete response.

## 2. Material and Methods

A single-institution database was queried for consecutive patients with LARC, biopsy-proven rectal adenocarcinoma, who underwent neoadjuvant CRT followed by elective radical surgery with TME with curative intent between January 1, 2003, and December 31, 2011.

Admission criteria were patients with rectal cancers located less than 12 cm distance anal verge and clinical stage T2N+M0 or cT3/4 N0/+M0.

Exclusion criteria were patients with other diagnosed neoplasia, short course RT, yp stage IV, R1/R2 surgery, and death within 60-day postoperative time.

All patients receiving neoadjuvant CRT who were operated within 8 weeks after radiotherapy conclusion were included in this analysis. Patients receiving short-course radiation were excluded since no downstaging occurs when immediate surgery is carried out.

Staging included rigid proctoscopy, total colonoscopy, chest, abdominal and pelvic CT scan, endorectal ultrasound (ERUS), pelvic magnetic resonance image (MRI) (since 2008), and carcinoembryonic antigen serum levels.

The neoadjuvant CRT protocol included a total irradiation of 50.4 Gy in 28 fractions and 5-fluorouracil by infusion pump.

Radical surgery was consisted mainly for sphincter saving rectal resection (SSRE) or abdominoperineal resection (APR) with TME. Regarding operative procedure selection, we considered the distance of the lesion to the anus, the comorbidities of the patient, and the condition of the anal sphincter.

Operated patients were subjected to adjuvant chemotherapy protocol for 6 months performed preferably with 5-fluorouracil (5-FU) or a combination 5-FU and oxaliplatinum.

**Figure 1:**
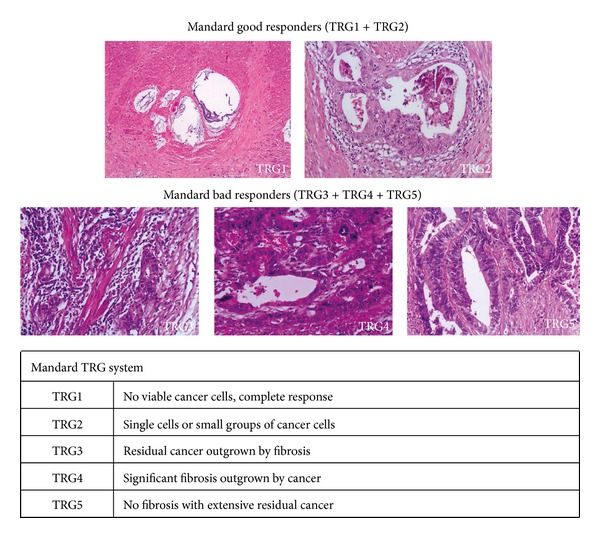
Mandard system.

**Figure 2:**
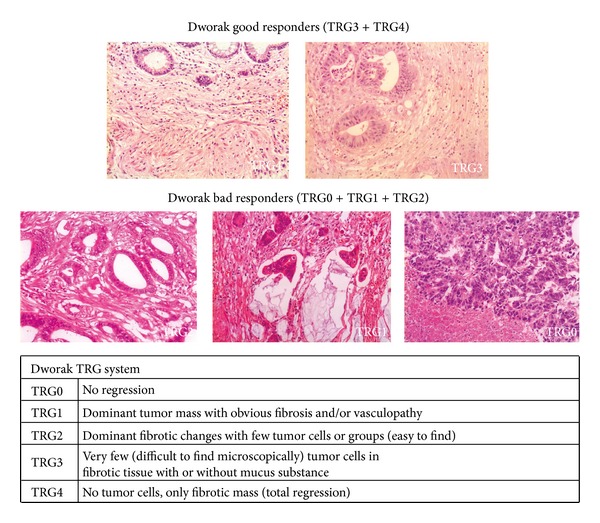
Dworak system.

Standard pathologic tumor staging of the resected specimen was performed in accordance with the guidelines of the American Joint Committee on Cancer (AJCC). Circumferential resection margin (CRM) was scored as positive when cancers cells were within 1 mm of the margin. Evidence of ypCR was defined as an absence of viable adenocarcinoma in the surgical specimen or the presence of lakes of mucus without tumor cells. The histology of all surgical specimens was reviewed and confirmed by an independent element and was classified based on two tumor regression grading systems: Mandard and Dworak (Figures 1 and 2).

### 2.1. Patients Were Divided in 4 Groups according to TRG

Mandard system, good responders were defined as Mandard TRG3+TRG4+TRG5; bad responders were defined as Mandard TRG1+TRG2.

Dworak system, good responders were defined as Dworak TRG3+TRG4; bad responders were defined as Dworak TRG2+TRG1+TRG0.

The groups in both systems (good responders versus bad responders) were used to evaluate outcome results.

Disease recurrence was evaluated according to location: locoregional (LR), systemic (DR), or mixed.

None of the patients were lost for followup.

All surviving patients were observed and their current status was confirmed.

### 2.2. Statistical Analysis

Mandard and Dworak TRG groups (good/bad) were compared in relation to age, sex, tumor distance from anal verge, clinical stage, surgical procedure performed, and pathological stage (yp stage) using the Student's *t*-student and the *X* ^2^.

Survival time was defined as the interval between the beginning of neoadjuvant therapy and the date of the last observation.

Oncologic outcomes were evaluated for overall survival (OS), disease-free survival (DFS), overall recurrence (OR), local recurrence (LR), and distant recurrence (DR).

Survival curves were performed using the Kaplan-Meier method and compared using the log-rank test. The influence of the covariates, Mandard TRG, Dworak TRG, ypN stage, ypT stage, and tumor distance from anal verge on the cumulative probability rates, was examined using the proportional hazard model (forward stepwise method) described by Cox.

*P* values are two-sided and *P* < 0.05 was considered significant. The statistical analysis was done using IBM SPSS Statistics version 20.

## 3. Results

The database query returned 157 patients. We excluded 18 patients: 11 patients with positive radial margin (R1 surgery), 3 patients yp stage IV, and four deaths within 60 days of postoperative period. The remaining 139 patients were evaluated.

The analysis of the clinical characteristics and surgery performed on the 139 patients can be seen in Table 1.

### 3.1. Surgery

Sphincter saving rectal resection with anastomosis (with or without protective ileostomy) was performed on 88 patients (63.3%). Abdominal-perineal resection was performed on 46 patients, and five patients were subjected to proctectomy with definitive stoma. The perioperative morbidity of the series was of 25% with 11 abdominal or pelvic abscesses, 2 anastomose leaks, 5 reoperations, and 2 readmissions.

### 3.2. Pathology

Stage distribution is shown in Table 2.

The average number of dissected lymph nodes in the surgical specimen was 8.2 (range 0–22). Circumferential resection margin >1 mm was confirmed in all 139 patients.

Response to neoadjuvant therapy is characterized in Table 1 with clinical parameters.

*TRG Classification*. The use of Mandard and Dworak systems allowed us to define two groups as previously mentioned: good responders (Mandard TRG1+2 or Dworak 3+4) and bad responders (Mandard TRG3+4+5 or Dworak TRG2+1+0).

Using Mandard system a good response to neoadjuvant CRT was attributed to 70 patients (50.4%) and a bad response was attributed to 69 patients (49.6%).

Using Dworak system a good response to neoadjuvant CRT was attributed to 54 patients (38.8%) and a bad response was attributed to 85 patients (61.2%).

The number of patients with a complete response (ypCR) is the same according to both systems (Mandard TRG1/Dworak TRG4 in 25–17.9%).

Table 2 shows T and N pathological staging according to Mandard and Dworak groups. Mandard TRG seems to better relate with ypN stage while Dworak TRG relates with ypT stage.

### 3.3. Disease Recurrence

#### 3.3.1. Pelvic Recurrence

Four patients (2.7%) had isolated pelvic recurrence. Among patients on the good response groups (Mandard and Dworak), pelvic recurrence appeared, 45 months after surgery, in 1 out of 70 (1.4%) and in 1 out of 54 (1.8%), respectively, patients with ypCR (Table 3).

#### 3.3.2. Distant Recurrence

Distant recurrence without pelvic recurrence appeared in 20 of the 139 patients (14.4%). If we consider only the patients with a good response, distant recurrence appeared in 6 out of 70 (8.6%) patients with “Mandard's good response” and in 6 out of 54 (11.1%) patients with “Dworak's good response.” For patients who had a complete pathologic response distant recurrence emerged in one patient only (brain metastasis 25 months after surgery). 

#### 3.3.3. Mixed Recurrence

Two patients (1.4%) had pelvic and distant disease. All were classified as bad responders according to Mandard and Dworak systems.

### 3.4. Survival

The mean follow-up was 56 months (range 6–125). Five years overall survival (OS) and five years disease free survival were 72.3% and 72.1%, respectively (Table 4).

**Figure 3:**
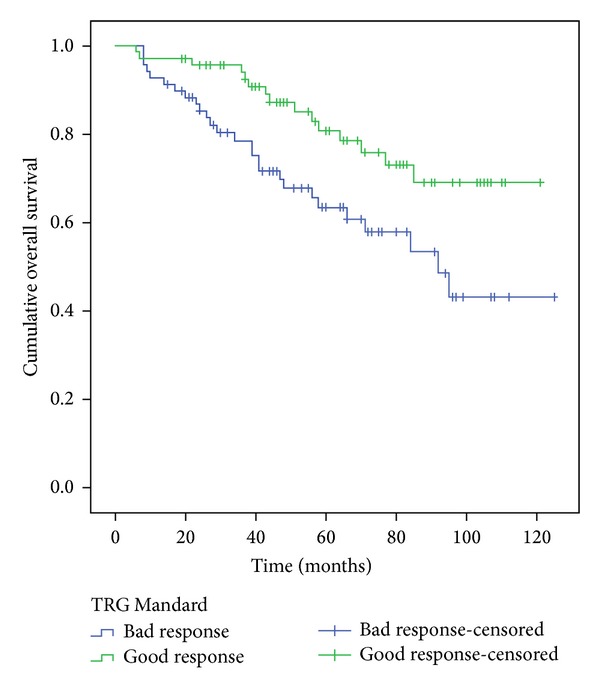
Five-year overall survival, comparison of the two groups Mandard.

**Figure 4:**
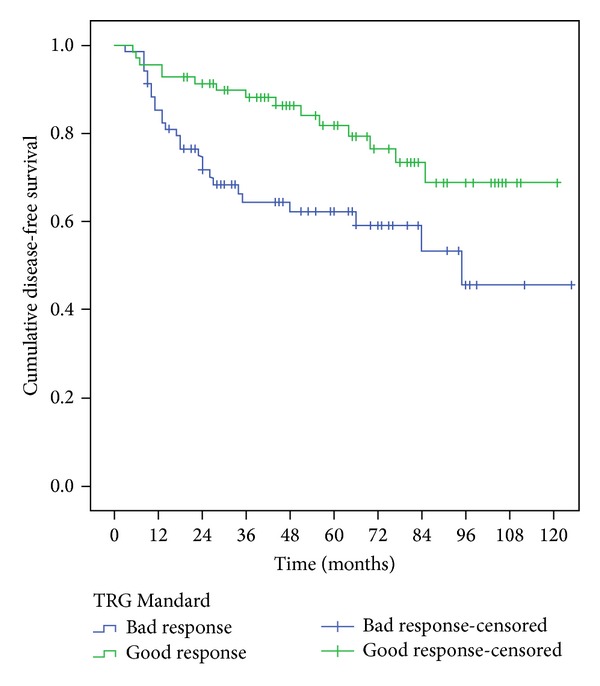
Five-year disease-free survival, comparison of the two groups Mandard.

The survival of patients who showed a good response on Mandard TGR was significantly higher than the ones with bad responses, according to 5-year overall survival (OS) and 5-year disease free survival (*P* = 0.013 and 0.007 resp.) as shown in Table 4 and Figures 3 and 4.

**Figure 5:**
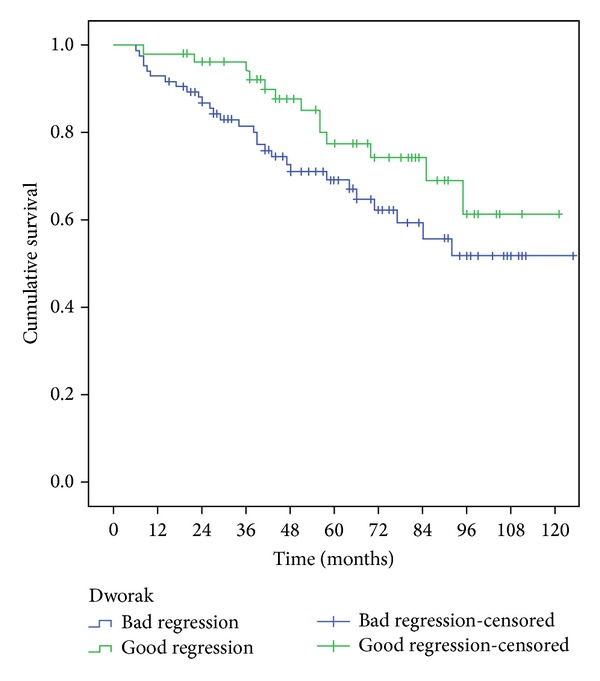
Five-year overall survival, comparison of the two groups Dworak.

**Figure 6:**
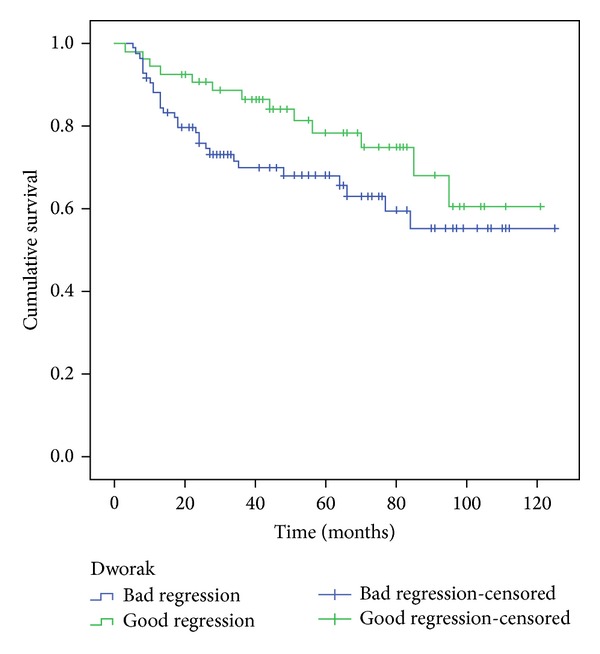
Five-year disease-free survival, comparison of the two groups Dworak.

Using Dworak system the results point in the same direction but without being statistically significant (according to the log-rank test, but not the Breslow test—Table 4 and Figures 5 and 6).

The two groups (good responders versus bad responders) on the two TGR (Mandard and Dworak) are statistically comparable with respect to age, sex, clinical stage, and surgical procedures performed with the exception of tumor distance from anal verge (*P* = 0.009/*P* = 0.02), ypN stage (ypN0/ypN+) (*P* =  <0.001/*P* = 0.001), and ypT stage (ypT0-2/ypT3-4) (*P* < 0.001/*P* < 0.001) (Tables 1 and 2). 

Cox regression model indicates that Mandard TRG is the only independent factor influencing overall survival (OS) and DFS after factoring in the following variables: Mandard TRG, Dworak TRG, ypN stage (ypN0/ypN+), ypT stage, and distance from anal verge (Table 5).

## 4. Discussion

Neoadjuvant CRT followed by TEM surgery is the widely accepted treatment for LARC [22–24]. Histologic changes after neoadjuvant CRT for rectal carcinoma vary considerably, with some entities showing complete absence of tumor cells, whereas others exhibit a mass of tumor cells with little or no regressive changes. Besides complete response, partial response can also be quantified. A method to assess treatment response is accomplished by grading histologic changes in the resected specimen that are caused by neoadjuvant CRT. Tumor regression grading has been proposed in a variety of classifications but their accuracy to correlate CRT induced histological changes with disease prognosis is not always present. Although there are no consensual pathological standards, Mandard and Dworak systems [12, 13] are broadly being used. For this reason we applied them in our studies. The former one essentially counts the number of residual tumor cells while the later one focuses on the quantification of fibrosis (Figures 1 and 2).

Both have 5 levels and are consistent in identifying a complete response (ypCR) but differences arise when the response is partial. Although early data suggest that the greatest degree of tumour regression is associated with the best clinical outcome, the clinical implication of moderate degrees of tumour regression is currently unclear.

In our studies the outcome for patients with a near complete response (Mandard TRG2 or Dworak TRG3) is almost similar to the outcome for patients with complete response (Mandard TRG1 or Dworak TRG4). This result suggests that it may be possible to combine tumors into a group of good responders (Mandard TRG1+2/Dworak TRG4+3) and a group of bad responders (Mandard TRG3+4+5/Dworak TRG2+1+0), since those who show significant histopathological regression and complete pathologic regression have a similarly better prognosis than the remaining poorly responding patients. This kind of division was also used by other authors [15, 19, 20, 25]. Our experience tells us that in defining a near complete response, Mandard TRG2 identifies a larger number of patients with better prognosis than Dworak TRG3. This finding may explain the better correlation between Mandard grading and disease prognosis, rather than with Dworak grading. Good responders have higher 5-year overall survival and 5-year disease free survival than bad responders, albeit these results are statistically significant only when Mandard system is considered. In our analysis a good response, defined as Mandard 1 and 2 classifications, was present in 70 of the 139 patients receiving CRT (50.4%).

However, the TRG cannot be considered as the only prognostic factor that affects rectal cancer patient's survival rate after chemoradiotherapy; sphincter saving surgery rate, CMR status, local recurrence rate, perineural invasion, and ypT and ypN stages are also thought to be crucial prognostic factors [7, 15, 26, 27]. In our series we analyzed those variables in the TRG groups created. The presence of a good response in either one of the two systems did not have an impact in terms of sphincter preservation surgery. When response was good in both TRG an estimated 78.5% reduction of positive lymph nodes was achieved when clinical values were compared (uN + = 28) against pathological ones (ypN + = 6). These aspects contributed to lowering locoregional and distant recurrence in good responders. It is possible that the impact of a good response in obtaining radial margins greater than 1 mm and the reduction of the number of positive lymph nodes and the pathological T stage have contributed in an effective way to increase the survival of this subgroup.

Mandard system, which shows the CRT effect, seems to be an additional prognostic factor that, along with TNM stage, predicts the survival and recurrence rates. A patient with a good response by Mandard is estimated to face a hazard ratio that is only 46% of the hazard faced by a patient who had a bad response (Table 5).

Some biases and limitations of our study can be pointed out: the series is small; the histology of all surgical specimens was reviewed retrospectively; the study protocol did not provide extra paraffin blocks from surgical specimen to confirm pCR diagnoses. The number of dissected lymph nodes, average 8.2 (0–22), can also be seen as a limitation. In order to better take advantage of our data, we intend to proceed with continued research in this area and to increase the number of patients enrolled in this study, maintaining as we have done so far: a single-institution database, a single and well-defined operative procedure, and the revision of all surgical specimens by an independent and experimented pathologist.

Finally, standardization of the Mandard TRG system and more research on the factors that affect the Mandard TRG need to be done. Clearly, the accuracy, reliability, and validity of the TRG system needs to be further investigated.

The clinical validation of a universally accepted regression scoring system is a key research priority.

## 5. Conclusion

The presence of tumor regression can be considered a prognostic factor. In terms of regression grades Mandard was the one that best correlated with the presence of therapeutic response and prognosis. The presence of a good response in terms of Mandard tumor regression grade was associated with a lower incidence of locoregional recurrence and improved survival.

## Figures and Tables

**Table 1:** Clinical parameters and *P* value of TRG groups.

Variables	*n* = 139	Mandard	Dworak
		Good versus bad response	Good versus bad response
		*P* value	*P* value
Sex			
Male	87 (62.6%)	0.52	0.64
Female	52 (37.4%)		
Age			
Mean (range)	64.2 (32–82)	0.12	0.34
Tumor distance from anal verge			
>6 cm	68 (48.9%)	**0.009 **	**0.02 **
≤6 cm	71 (51.1%)		
Clinical stage			
II	77 (53.5%)	0.13	0.49
III	67 (46.5%)		
Surgical procedure			
SSRR (sphincter saving rectal resection)	88 (63.3%)	0.18	0.33
APR (abdominoperineal resection)	46 (33.1%)		
Other (rectal resection without anastomose)	5 (3.6%)		

**Table 2:** Comparison between TRG and pathological variables.

Variables	*n* (%)	Mandard good response *n* (%)	Mandard bad response *n* (%)	*P* value	Dworak good response *n* (%)	Dworak bad response *n* (%)	*P* value
ypT stage							
T (0–2)	88 (66.1)	42 (60)	7 (10.1)	**<0.001 **	36 (66.7)	13 (15.3)	**<0.001 **
T (3-4)	56 (38.9)	28 (40)	62 (89.9)		18 (33.3)	72 (84.7)	
ypN stage							
N0	5 (3.5)	61 (87.1)	36 (52.2)	**<0.001 **	47 (87)	50 (58.8)	**0.001 **
N (1-2)	139 (96.5)	9 (12.9)	33 (47.8)		7 (13)	35 (41.2)	

**Table 3:** Clinical long-term outcome.

	*n* (%)	Mandard good response *n* (%)	Mandard bad response *n* (%)	Dworak good response *n* (%)	Dworak bad response *n* (%)
Overall recurrence disease	26 (18.7)	7 (10)	19 (27.5)	7 (12.9)	19 (22.3)
Local	4 (2.9)	1 (1.4)	3 (4.3)	1 (1.8)	3 (3.5)
Distant	20 (14.4)	6 (8.6)	14 (20.3)	6 (11.1)	14 (16.4)
Local and distant	2 (1.4)	0 (0)	2 (2.9)	0 (0)	2 (2.3)

**Table 4:** Clinical long-term outcome, survival percent.

	(%)	Mandard good response (%)	Mandard bad response (%)	*P* value	Dworak good response (%)	Dworak bad response (%)	*P* value
Five-year overall survival (OS)	72.3	79.5	60.7	**0.013** ^∙^	77.4	69.1	0.10^∙^
	(se = 4.2)	(se = 5.4)	(se = 6.3)	**0.010** ^∙∙^	(se = 6.4)	(se = 5.5)	**0.047** ^∙∙^

Five-year disease free survival (DFS)	72.1	81.7	61.7	**0.007** ^∙^	78.4	68.1	0.10^∙^
	(se = 4.1)	(se = 5.1)	(se = 6.3)	**0.004** ^∙∙^	(se = 6.2)	(se = 5.4)	**0.047** ^∙∙^

^∙^Log rank test; ^∙∙^Breslow test.

**Table 5:** Survival in patients Mandard TRG(1+2) versus Mandard TRG(3+4+5), the only significant covariable retained in the multivariable analysis tested by the likelihood ratio test (stepwise forward model).

	Hazard ratio (95% confidence interval)	*P* value
OS	0.46 (0.24–0.86)	**0.016 **
DFS	0.34 (0.23–0.81)	**0.009 **
